# Action Mechanism of Fibroblast Growth Factor-2 (FGF-2) in the Promotion of Periodontal Regeneration in Beagle Dogs

**DOI:** 10.1371/journal.pone.0131870

**Published:** 2015-06-29

**Authors:** Toshie Nagayasu-Tanaka, Jun Anzai, Shu Takaki, Noriko Shiraishi, Akio Terashima, Taiji Asano, Takenori Nozaki, Masahiro Kitamura, Shinya Murakami

**Affiliations:** 1 Pharmacology Department, Drug Research Center, Kaken Pharmaceutical Co., LTD., Kyoto, Kyoto, Japan; 2 Department of Periodontology, Osaka University Graduate School of Dentistry, Suita, Osaka, Japan; Department of Biomaterials, JAPAN

## Abstract

Fibroblast growth factor-2 (FGF-2) enhances the formation of new alveolar bone, cementum, and periodontal ligament (PDL) in periodontal defect models. However, the mechanism through which FGF-2 acts in periodontal regeneration *in vivo* has not been fully clarified yet. To reveal the action mechanism, the formation of regenerated tissue and gene expression at the early phase were analyzed in a beagle dog 3-wall periodontal defect model. FGF-2 (0.3%) or the vehicle (hydroxypropyl cellulose) only were topically applied to the defect in FGF-2 and control groups, respectively. Then, the amount of regenerated tissues and the number of proliferating cells at 3, 7, 14, and 28 days and the number of blood vessels at 7 days were quantitated histologically. Additionally, the expression of osteogenic genes in the regenerated tissue was evaluated by real-time PCR at 7 and 14 days. Compared with the control, cell proliferation around the existing bone and PDL, connective tissue formation on the root surface, and new bone formation in the defect at 7 days were significantly promoted by FGF-2. Additionally, the number of blood vessels at 7 days was increased by FGF-2 treatment. At 28 days, new cementum and PDL were extended by FGF-2. Moreover, FGF-2 increased the expression of bone morphogenetic protein 2 (BMP-2) and osteoblast differentiation markers (osterix, alkaline phosphatase, and osteocalcin) in the regenerated tissue. We revealed the facilitatory mechanisms of FGF-2 in periodontal regeneration *in vivo*. First, the proliferation of fibroblastic cells derived from bone marrow and PDL was accelerated and enhanced by FGF-2. Second, angiogenesis was enhanced by FGF-2 treatment. Finally, osteoblastic differentiation and bone formation, at least in part due to BMP-2 production, were rapidly induced by FGF-2. Therefore, these multifaceted effects of FGF-2 promote new tissue formation at the early regeneration phase, leading to enhanced formation of new bone, cementum, and PDL.

## Introduction

Regeneration of periodontal tissue is the ultimate goal of periodontal therapy. However, no conventional periodontal and/or surgical treatment can regenerate lost periodontal tissue or its functionality. To address this limitation, various new regenerative therapies have been introduced, such as guided tissue regeneration (GTR) treatment [1] and the application of an enamel matrix derivative [2], and some success has been achieved in periodontal tissue regeneration.

Moreover, a variety of recombinant cytokine have been investigated in terms of their ability to stimulate periodontal tissue regeneration [3–7]. We have been investigating the induction of periodontal regeneration by topical application of fibroblast growth factor-2 (FGF-2). FGF-2 facilitates fibroblastic cell proliferation, angiogenesis [8], and bone formation [9, 10]. We have demonstrated that FGF-2 enhances the formation of new alveolar bone, cementum, and periodontal ligament (PDL), resulting in the induction of balanced periodontal tissue regeneration in artificial periodontal defect models in the beagle dog [11, 12] and non-human primates [13]. We then conducted clinical trials of 2- and 3-wall vertical bone defects in patients with periodontitis and demonstrated that FGF-2 showed significant superiority over the vehicle alone in terms of the percentage of bone fill in modified Widman periodontal surgery [14, 15]. Furthermore, FGF-2 in combination with a collagen gel, GTR therapy, or beta-tricalcium phosphate enhances periodontal regeneration in dogs [16–18]. Collectively, it has been demonstrated that FGF-2 enhances the quantity of regenerated tissues. However, the detailed mechanism underlying FGF-2 in periodontal regeneration *in vivo* has not been fully clarified yet.

In this study, to reveal the action mechanism of FGF-2 in periodontal regeneration, we conducted two investigations using a beagle dog 3-wall periodontal defect. First, we quantitated the amount of newly formed tissues as well as angiogenesis and the number of proliferating cells at the early regenerative phase. Second, we analyzed the osteogenic gene expression in the regenerated tissue of the defect. In consequence, we revealed that FGF-2 stimulated proliferative responses and angiogenesis and increased the expression of several osteogenic differentiation markers in the regenerated tissue.

## Methods

### Preparation of test substances

A solution of 0.3% FGF-2 was prepared by dissolving freeze-dried human recombinant FGF-2 (Kaken Pharmaceutical Co. Ltd) in a 3% hydroxypropyl cellulose (HPC) solution. The vehicle was the 3% HPC solution alone. HPC was added to facilitate administration because of its high viscosity. Either the vehicle or 0.3% FGF-2 solution was administered to the control and FGF-2 groups, respectively.

### Animals

From the viewpoint of animal welfare, we believe that the lives of laboratory animals must be respected and their distress minimized, conducting scientifically rational experiments with the minimum number of animals. TOYO beagle dogs were obtained from Kitayama Labes Co., Ltd (Nagano, Japan). Sixteen female beagle dogs (59–68 months old, 11.0–14.3 kg) were used for the histological and quantitative analyses of proliferating cell nuclear antigen (PCNA)-positive cells in the defect. Four female dogs (62–69 months old, 9.4–10.7 kg) were used to calculate the blood vessel area. Twelve females (60–69 months old, 9.2–13.2 kg) were used for real-time polymerase chain reaction (PCR) analyses. These 12 dogs were also applied to other examinations after tissue collection.

They were housed individually and moved freely in stainless steel cages under conditions at 18–26°C temperature, 30–70% humidity, and 12 h lighting (07:00–19:00). Animal room and cage cleaning were performed daily. In addition, during the cage cleaning, the dogs were taken outside the cage to reduce their stress. Animals were provided with 230 g of solid food (LABO D STOCK; Nihon Nosan Kogyo Co., Yokohama, Japan) per day and filtered tap water *ad libitum*. This study was approved by the Animal Experiments Ethics Committee of Kaken Pharmaceuticals Co., Ltd [Permit Number: K08-290 (Date of approval: Oct 28, 2008) and K12-383 (Date of approval: Dec 7, 2012)].

Kaken follows in-house regulations in complying with ‘Japan's Act on Welfare and Management of Animals’ and the international and domestic related guidelines. The ethics committee established on the basis of the in-house regulations reviews whether all animal experimental protocols are prepared based on "3Rs (Replacement, Reduction and Refinement) principles" in advance, and implements self-inspections and assessments of the animal experiment processes and the facility operations. In addition, Kaken has been certified as a qualified institution for Laboratory Animal Care and Use by The Japan Health Sciences Foundation (Tokyo, Japan), which assesses and verifies compliance to the ‘Basic Guidelines for Proper Conduct of Animal Testing and Related Activities in the Research Institutions under the jurisdiction of the Ministry of Health, Labour and Welfare (Japan)’ as an external organization.

### Periodontal surgery and administration of test substances

All beagle dogs were subjected to an artificially created 3-wall periodontal defect. After the dogs were anesthetized subcutaneously with xylazine (0.4 mg/dog; Celactal: Bayer Yakuhin, Ltd., Tokyo, Japan), intravenously with pentobarbital (10 mg/kg), and intragingivally with 2% lidocaine and 0.00125% adrenaline (Xylocaine: Dentsply-Sankin K.K., Tokyo, Japan), the right and left fourth premolars in the mandible were extracted. For pain relief, meloxicam (0.2 mg/kg; Metacom: Boehringer Ingelheim Vetmedica, Inc., MO, US) were administered subcutaneously. Three months later, the beagle dogs were anesthetized as described above, a mucoperiosteal flap was raised, and 3-wall periodontal defects (mesio-distal width × buccolingual width × depth: 5 × 3 × 4 mm) were surgically created on the mesial portion of the mandibular first molar on both sides. The supporting bone and cementum of the teeth were removed with steel burs and the exposed root surfaces of the teeth were smoothened using Gracey curettes. Either the vehicle or 0.3% FGF-2 solution (60 μL/site) was then administered to the defects. The gingival flaps were sutured, and penicillin (40,000 U/dog) and streptomycin (200 mg/dog) were administered subcutaneously to prevent infection after surgery. Animals were given about 400 g of soft food (Pedigree: Mars Japan Limited, Tokyo, Japan) per day during the first 7 postoperative days.

### Histological analysis

Under general anesthesia, four beagle dogs each were euthanized by exsanguination at 3, 7, 14, and 28 days after application. Tissues containing the test tooth and defect region on both sides were extracted and fixed with neutral-buffered 10% formalin. After decalcification, tissues were embedded in paraffin using common methods. Serial sections were cut in the mesio-distal plane and stained with Azan and Hematoxylin-Eosin (HE).

Histometric measurements were performed using WinRoof image analysis software (ver. 5.03, Mitani Co., Tokyo, Japan) to evaluate the amount of regenerated tissue. The number of blood vessels in the mesio-distal 1 mm width of the connective tissue on the root surface at 7 days was counted with the aid of an ocular grid mounted on an eyepiece of a light microscope.

### Immunohistochemistry and quantitative analysis of PCNA-positive cells

Sections were immunostained with a rabbit polyclonal anti-human PCNA antibody (Santa Cruz Biotechnology Inc., Dallas, Texas, USA) using a Histofine SAB-PO (R) kit (Nichrei Bioscience, Inc., Tokyo, Japan) according to the manufacturer’s protocol. For immunostaining of collagen type 1, a mouse monoclonal anti-collagen type 1 antibody (Abcam, Cambridge, UK) and Histofine SAB-PO (M) kit (Nichrei Bioscience, Inc., Tokyo, Japan) were used. The reaction products were then developed using diaminobenzidine (DAB) (Dako, Glostrup, Denmark) and counterstained using hematoxylin.

To quantitate the proliferating cells, the number of PCNA-positive cells was counted by the same examiner (TNT) with the ocular grid in a blinded manner. In the bone defect area, the defect was compartmentalized into four zones (outside, margin, center, and upper). Based on the ratio of each zone to the defect, we evaluated 6 mm^2^ in the margin, 1 mm^2^ in the center, and 3 mm^2^ in the upper per defect. A total of 4 mm^2^ in the outside zone was selected around the defect corner, the existing PDL, and the superior border of the existing bone. The number of positive cells in each zone was indicated as the mean per 1 mm^2^. On the root surface, the total number of positive cells in a 0.25 mm width was counted from the defect bottom to the apical based on the width of the existing PDL.

For double immunostaining, sections were first immnostained with the anti-PCNA antibody using a Histofine Simple Stain AP (R) kit (Nichrei Bioscience, Inc., Tokyo, Japan) according to the manufacturer’s protocol. Permablue (Diagnostic Biosystms, CA, US) was used as the chromogen. Then, the sections were immnostained with a mouse monoclonal anti-vimentin antibody (Covance, US) using Histofine SAB-PO (M) kit. DAB was used as the chromogen. Negative controls were performed by replacing the respective primary antibodies with isotype and concentration-matched irrelevant antibodies. In addition, the patterns of double staining were carefully compared with those of single staining.

### Blood vessel area in the defect

Four beagle dogs were euthanized by exsanguination at 7 days after application under general anesthesia. After exsanguination, saline and then Indian ink with 3% gelatin were perfused into the carotid artery. Tissues were fixed and decalcified as described above. Mesio-distal sections of specimens were cut and stained with eosin. The areas of black ink and the defect were measured using WinRoof image analysis software. The percentage of the blood vessel area in the defect was calculated by the area of black ink / the defect area × 100.

### Real-time PCR

Six dogs each were anesthetized at 7 and 14 days after application. Under general anesthesia, the regenerated tissues in the defect on both sides were harvested. The collected tissues were mixed with ISOGEN (Nippon Gene Co., Ltd., Tokyo, Japan) to isolate total RNA. After the removal of genomic DNA using a DNA-Free RNA Kit (ZYMO RESEARCH Corp., Irvine, CA, USA), first-strand cDNA was synthesized from 1 μg total RNA using a High-Capacity cDNA Reverse Transcription Kit with RNase Inhibitor (Life Technologies Corp., Carlsbad, CA, USA). Quantitative PCR analysis was performed in an ABI PRISM 7000 Sequence Detection System (Life Technologies) under the following conditions: 50°C for 2 min and 95°C for 10 min, and then 45 cycles of 95°C for 15 sec and 60°C for 1 min. Primer/probe sets were provided by TaqMan Gene Expression Assays (Life Technologies); Bone morphogenetic protein 2 (BMP-2) (Cf02695364_s1), Sp7 transcription factor (osterix) (Cf02679117_s1), alkaline phosphatase (ALP) (Cf02623585_m1), osteocalcin (OC) (Cf02623891_g1), 18S ribosomal RNA (18S rRNA) (Hs99999901_s1). 18S rRNA was used as an internal control. Gene expression was calculated by the standard curve method for relative quantitation. The expression of target genes represents the ratio relative to the mean of the control group at 7 days.

### Statistical analysis

The mean and standard deviation (SD) were calculated for each measurement, and the differences between treatments were analyzed by the t-test or linear mixed model. The level of significance was set at 5%. Statistical analyses were performed using SPSS for Windows software (t-test, ver 14; SPSS, Chicago, IL, USA) and SAS (linear mixed model, ver. 9.2; SAS Institute Japan, Japan).

## Results

### Transition of regenerated tissue in the bone defect

**Fig 1:**
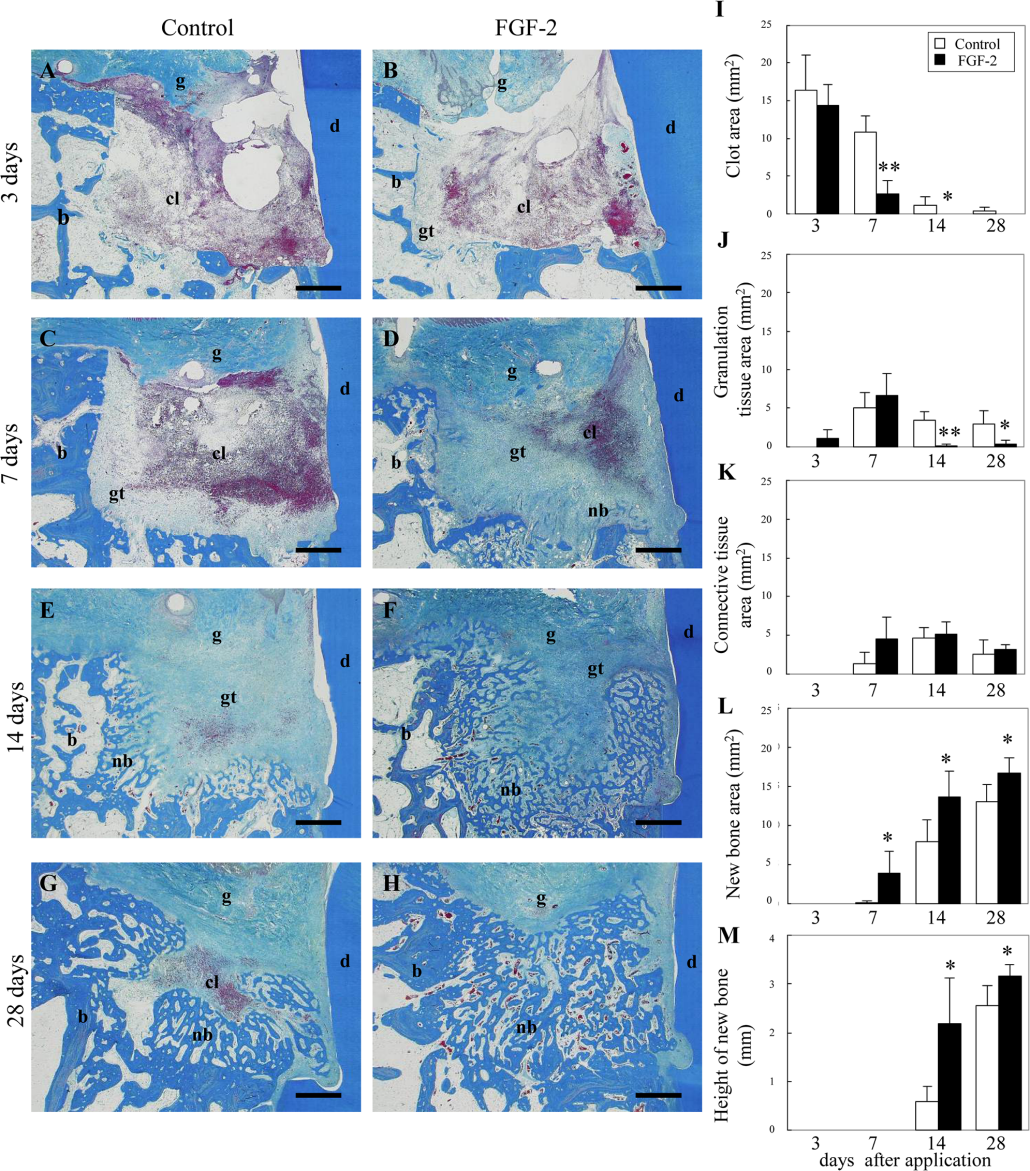
The transition of the histological overview in the 3-wall periodontal defect. (A–H): Representative photomicrographs are shown at 3 days (A, B), 7 days (C, D), 14 days (E, F), and 28 days (G, H) after application. The control (vehicle only) (A, C, E, G) or FGF-2 (B, D, F, H) were applied to defects. Scale bar represents 1 mm. cl, clot; gt, granulation tissue; nb, new bone; b, existing bone; d, dentin; g, gingival connective tissue. (I–M): Histological analysis of tissue regeneration in the bone defect. The areas of the clot (I), granulation tissue (J), connective tissue (K), and new bone (L) were measured in the bone defect. The height of new bone (M) was measured on the near side of the root surface. All results are presented as the mean ± SD (n = 4) (*, p <0.05; **, p<0.01 compared with the control, t-test).

Histologically, we observed (Fig 1A–1H) and quantitated regenerated tissue (Fig 1I–1M) in bone defects.

At 3 days after application, a blood clot with infiltration of inflammatory cells filled most of the defect in FGF-2 and control groups (Fig 1A, 1B and 1I). However, in the FGF-2 group, fibroblastic cells were expanded from the existing bone. The area of the granulation tissue in the FGF-2 group was larger than that in the control group (Fig 1J). Angiogenesis was observed in the newly formed granulation tissue. At 7 days, in the control group, the bone defect was largely occupied by the clot and partially by granulation tissues (Fig 1C). In the FGF-2 group, as the area of the clot diminished, granulation and connective tissues were formed across the bone defects (Fig 1D and 1K). Considerable amounts of new bone formation were then observed at the margin zone of the defect. The bone area in the FGF-2 group was significantly larger than that in the control group (Fig 1L). At 14 and 28 days, in the control group, a mixture of granulation tissue, connective tissue, and new bone filled in the bone defect (Fig 1E and 1G). In the FGF-2 group, new bone was formed in almost all parts of the bone defect (Fig 1F and 1H). The area and height of the new bone in the FGF-2 group was significantly larger than those in the control group (Fig 1M).

Thus, FGF-2 promoted granulation tissue formation accompanied by angiogenesis. This granulation tissue was rapidly replaced by new bone. As a result, FGF-2 promoted the bone formation.

### Transition of regenerated tissue on the root surface

**Fig 2:**
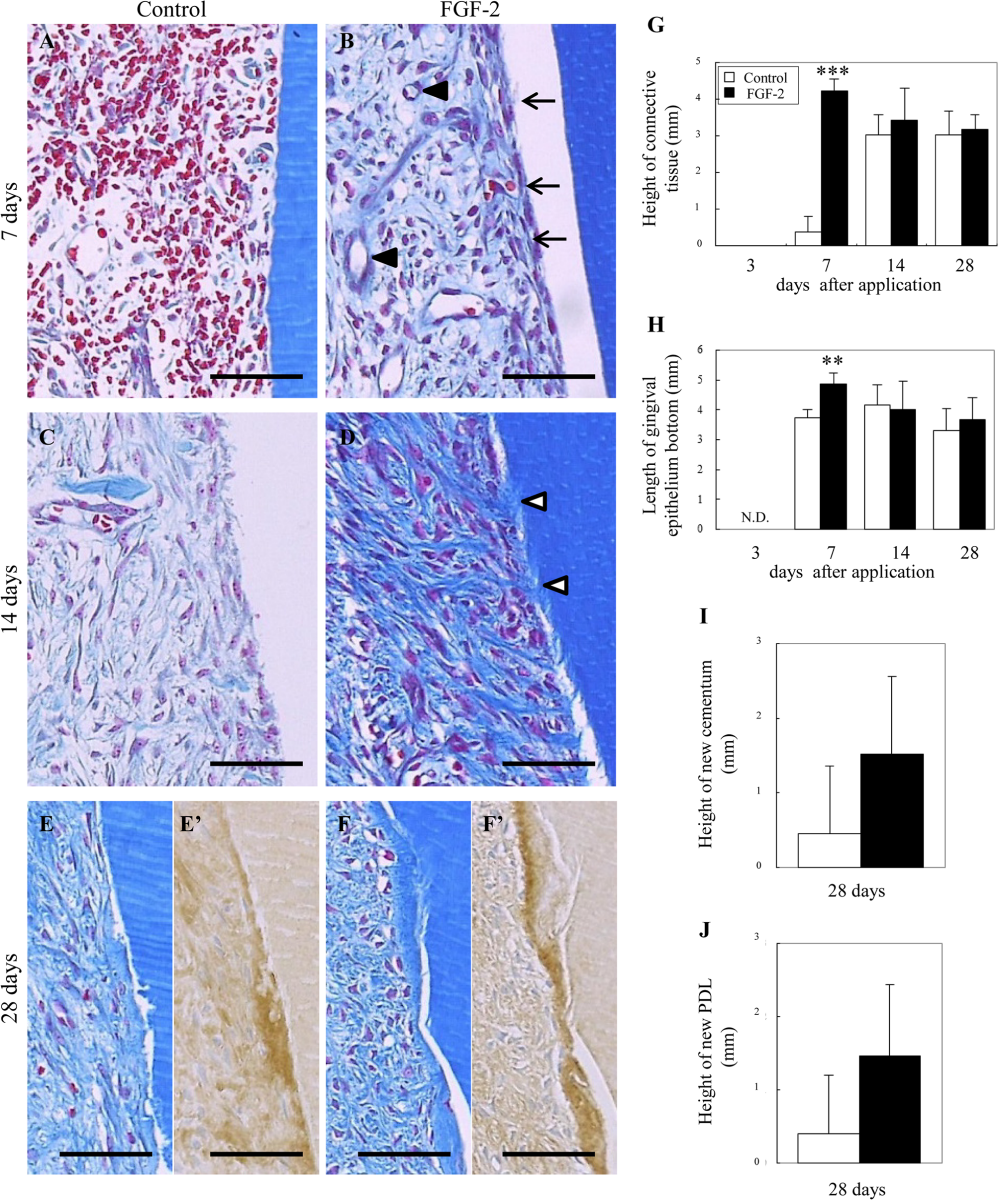
Histology of the regenerated tissue on the root surface. (A–F): Representative photomicrographs from control (A, C, E) and FGF-2 (B, D, F) groups are shown at 7 days (A, B), 14 days (C, D) and 28 days (E, F). Immunostaining of collagen type 1 in E and F are shown in E’ and F’. Arrow, thickly lined by the fibroblastic cells; black arrowhead, neovessel; white arrowhead, Sharpey’s fiber. Scale bar represents 50 μm. (G–J): Histological analysis of tissue regeneration on the root surface. (G): Height of the newly connective tissue from the defect bottom. (H): Length of the gingival epithelium from the defect bottom. N.D., no gingival tissue contacted the root surface. (I, J): Height of the new cementum and PDL from the defect bottom at 28 days. All results are presented as the mean ± SD (n = 4) (**, p<0.01; ***, p <0.001 compared with the control, t-test).

We evaluated the regenerated tissue on the root surface (Fig 2A–2F), and the height of the regenerated tissue and the length of the gingival epithelium from the defect bottom (Fig 2G–2J).

At 3 days, a clot covered the root surface in both FGF-2 and control groups. However, in the FGF-2 group, a few fibroblastic cells were expanded from the existing PDL. At 7 days, the clot was covered but some connective tissues were extended in the control group (Fig 2A and 2G). More pronounced tissue masses of fibroblastic cells were extended to the whole region of the root surface in the FGF-2 group (Fig 2B). Compared with the control, the height of the connective tissue was significantly increased by FGF-2 treatment. Interestingly, on the superficial layer, tightly lined fibroblastic cells were coronally stacked from the bottom (Fig 2B, arrows). In this connective tissue on the root surface, we observed marked angiogenesis (Fig 2B, black arrowheads). Compared with the control, the length of the epithelium from the defect bottom was significantly maintained at a high level by FGF-2 (Fig 2H). At 14 days, the new connective tissue was covered entirely in the control group, but the new tissues with a poor collagen density were immature (Fig 2C). Connective tissues with tightened collagen fibers were extended in the FGF-2 group (Fig 2D). Some of these collagen fibers connected the new partially-formed cementum with the alveolar bone, revealing the formation of new PDL with Sharpey’s fibers (Fig 2D, white arrowheads). At 28 days, in the control group, thin new cementum formation with new PDL was observed in one of four samples (Fig 2E). In the FGF-2 group, new cementum formation with new PDL was observed in three of four samples (Fig 2F). This new cementum was stained intensely for collagen type 1 (Fig 2E’ and 2F’). The amount of new cementum and PDL in the FGF-2 group was higher than that in the control group (Fig 2I and 2J). No ankylosis was observed in either group during the experiment.

Thus, the early formation of connective tissue induced by FGF-2 maintained the height of the gingival tissue at a high level, creating a regenerative space and eventually promoting the regeneration of new cementum and PDL.

### Localization and quantitation of proliferating cells in the bone defect

**Fig 3:**
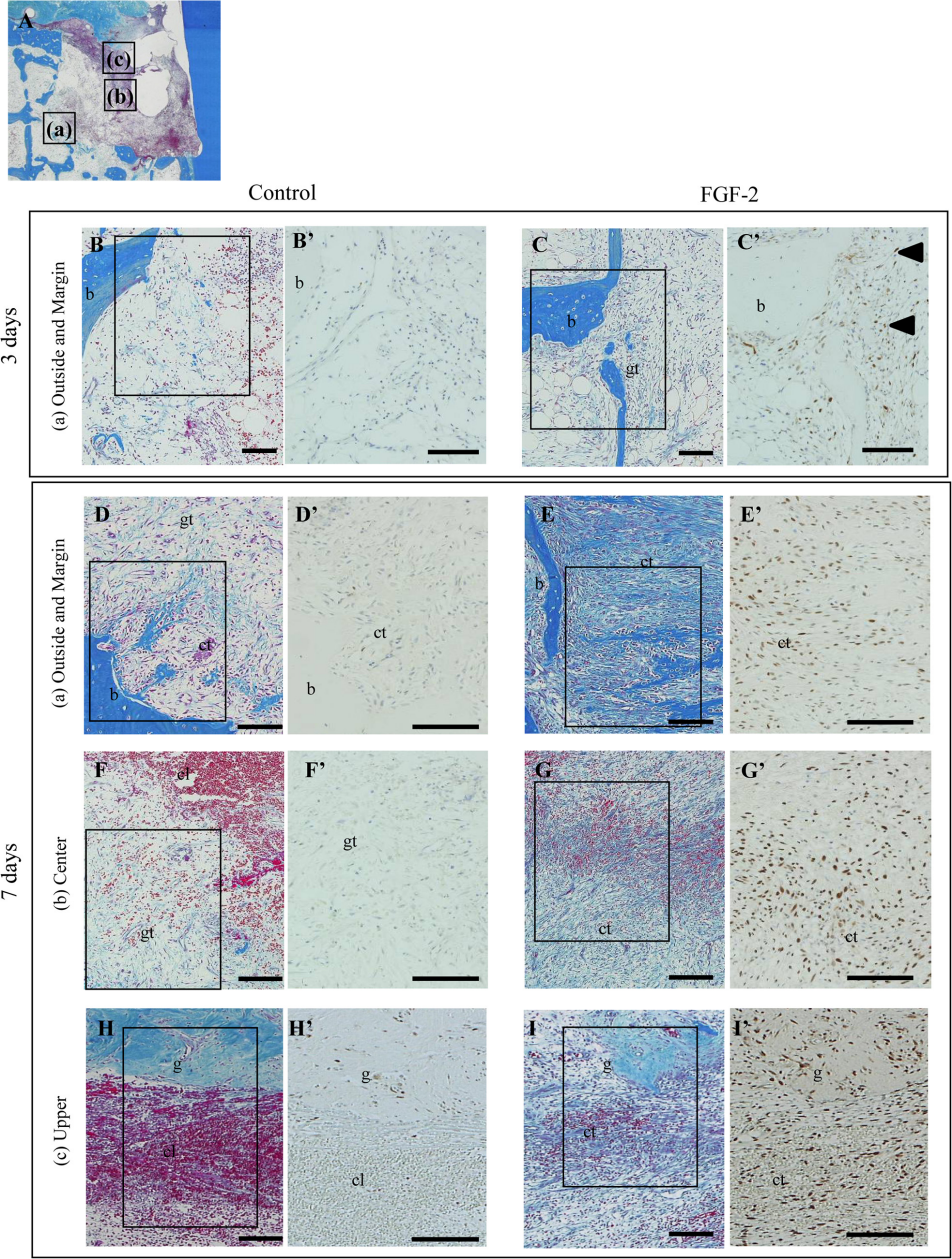
Localization of proliferating cells in the bone defect. (A): Schematic of the observation regions; outside and margin zones (a), center zone (b), and upper zone (c) of the defect. (B–I): Representative histological overviews from control (B, D, F, H) and FGF-2 (C, E, G, I) groups are shown at 3 days (B, C) and 7 days (D–I). Immunostaining of PCNA and higher magnifications of the rectangular areas in B–I are shown in B’–I’. cl, clot; gt, granulation tissue; ct, connective tissue; b, existing bone; g, gingival connective tissue; black arrowhead, positive cells expanded from the existing bone marrow. Scale bar represents 100 μm.

**Fig 4:**
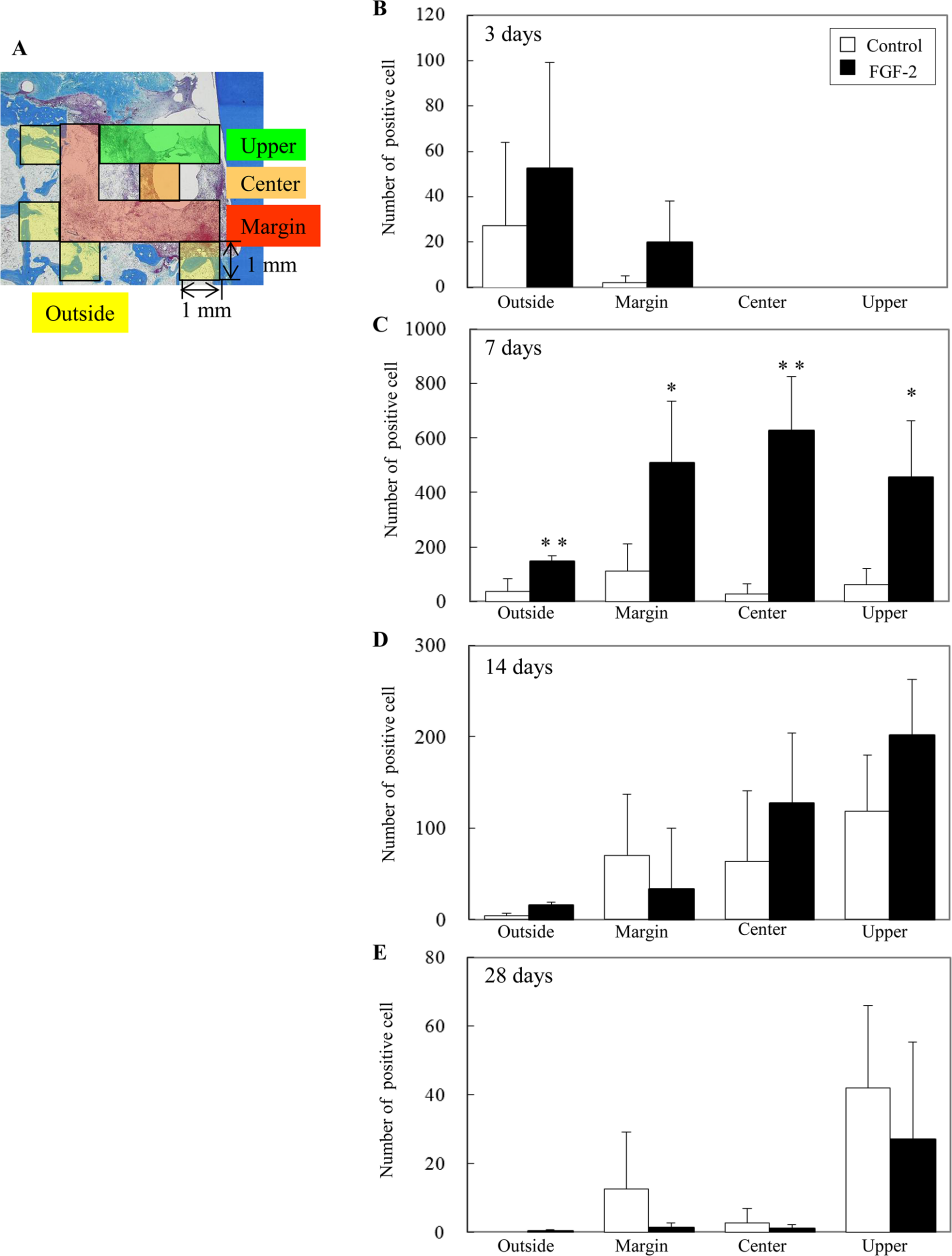
Numbers of proliferating cells in the bone defect. (A): Schematic of the measurement regions. The defect was compartmentalized into four zones: outside, margin, center, and upper. We evaluated 4 mm^2^ in the outside zone, 6 mm^2^ in the margin zone, 1 mm^2^ in the center zone, and 3 mm^2^ in the upper zone per defect. (B–E): Y-axis values represent the number of PCNA-positive cells per 1 mm^2^. All results are presented as the mean ± SD (n = 4) (*, p <0.05; **, p <0.01 compared with the control, t-test).

To reveal an active proliferation zone, PCNA-positive cells were quantitated in each zone (outside, margin, center, and upper) of the bone defect (Figs 3 and 4).

At 3 days, a few PCNA-positive cells were observed among the bone marrow cells of the outside zone in the control group (Fig 3B’). In the FGF-2 group, some positive cells were found among the bone marrow cells of the outside zone and the fibroblastic cells extending from the existing bone marrow in the margin zone (Fig 3C’, arrowheads). Compared with the control group, the number of the positive cells in the FGF-2 group was 2.5-fold higher in the outside zone and 10-fold higher in the margin zone (Fig 4B). At 7 days, the majority of positive cells was observed in the margin zone of the control group (Fig 3D’, 3F’ and 3H’), but at a high density in all zones of the FGF-2 group (Fig 3E’, 3G’ and 3I’). Additionally, quantitative data showed that the number of positive cells in all zones of the FGF-2 group was significantly higher than that of the control group (Fig 4C). These PCNA-positive cells expressed vimentin (S1 Fig). At 14 days, most of the positive cells were found in the center and upper zones of both groups (Fig 4D). The peak number of positive cells was at 14 days in the control group but at 7 days in the FGF-2 group. In a comparison of the maximum number of positive cells in both groups, the number of positive cells in the FGF-2 group at 7 days was markedly higher than that in the control group at 14 days. At 28 days, in both groups, positive cells were mostly observed in the upper zone (Fig 4E). The number of positive cells in the FGF-2 group was lower than that in the control in all zones.

Thus, these findings show the transition of newly divided cells from the existing bone to the margin, center, and upper zones of the bone defect. We also found that FGF-2 shifted the proliferative phase to earlier and enhanced the proliferative activity in the bone defect. The bone defect was promptly filled with proliferating cells, resulting in promotion of the regenerated tissue formation.

### Localization and quantitation of proliferating cells on the root surface

**Fig 5:**
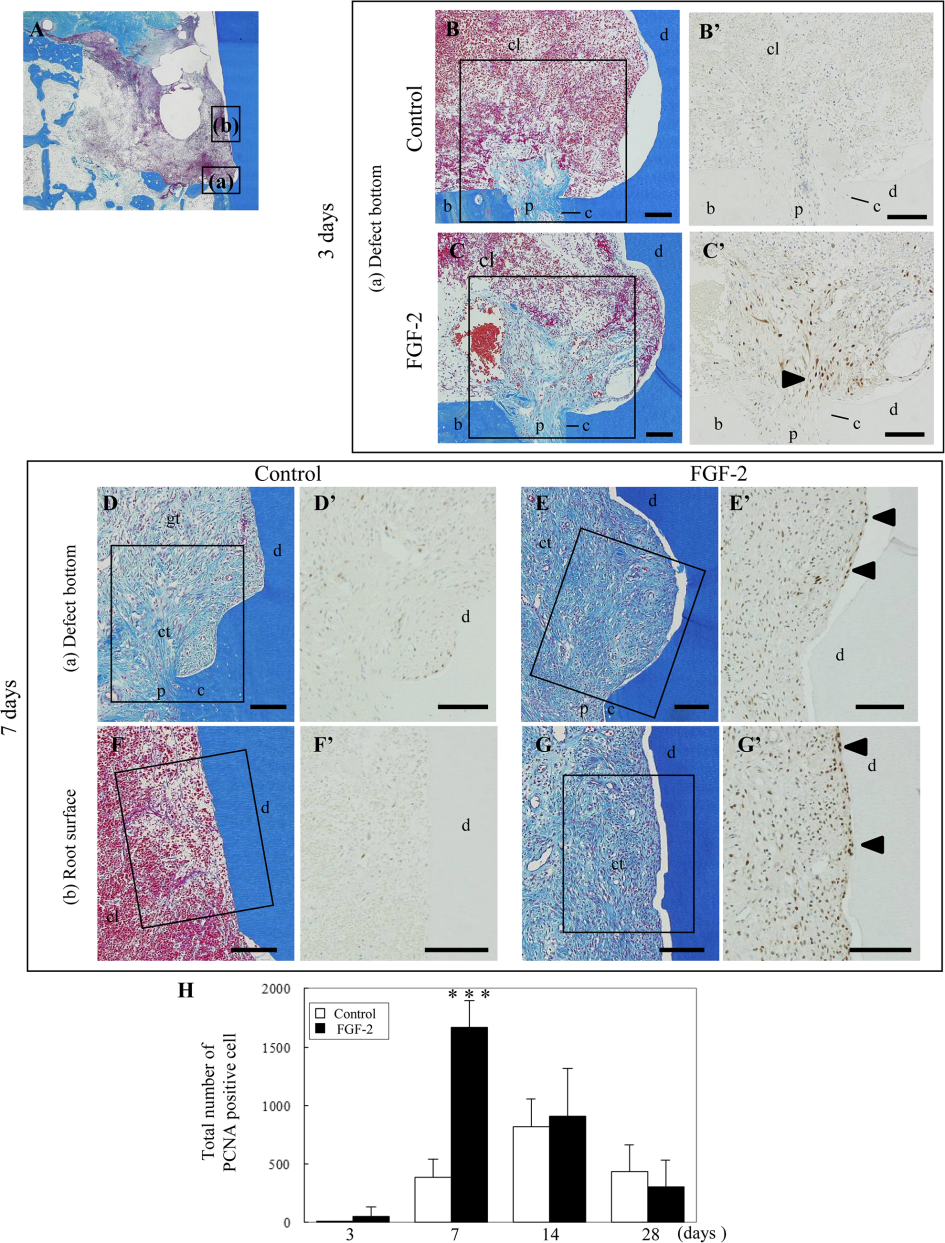
Localization of proliferating cells on the root surface. (A): Schematic of the observation regions; defect bottom around the existing PDL (a) and root surface (b). (B–G): Representative histological overviews from control (B, D, F) and FGF-2 (C, E, G) groups are shown at 3 days (B, C) and 7 days (D–G). Immunostaining of PCNA and higher magnifications of the rectangular areas in B–G are shown in B’–G’. cl, clot; gt, granulation tissue; ct, connective tissue; b, existing bone; d, dentin; p, existing PDL; c, cementum; arrowheads, positive cells extending from the existing PDL. Scale bar represents 100 μm. (H): Total number of proliferating cells in a 0.25 mm width from the root surface. The number of PCNA-positive cells was counted from the defect bottom to the apical on the root surface. The results are presented as the mean ± SD (n = 4) (***, p <0.001 compared with the control, t-test).

To examine the effect of FGF-2 on cell proliferation, the location of PCNA-positive cells on the root surface was observed at 3 and 7 days (Fig 5B’–5G’). Furthermore, the number of PCNA-positive cells was quantitated in a 0.25 mm width from the root surface (Fig 5H).

At 3 days, no PCNA-positive cells were observed in the control group (Fig 5B’). Some positive cells among the fibroblastic cells extending from the existing PDL were found in the FGF-2 group (Fig 5C’, arrowhead). At 7 days, positive cells were mainly observed around the existing PDL in the control group (Fig 5D’ and 5F’). In the FGF-2 group, positive cells were expanded coronally along the root surface (Fig 5E’ and 5G’). In particular, a row of vertical positive cells was observed on the superficial layer (Fig 5E’ and 5G’, arrowhead). The number of positive cells in the FGF-2 group was significantly increased by 4-fold compared with that in the control (Fig 5H). These PCNA-positive cells expressed vimentin (S2 Fig). At 14 and 28 days, the number of positive cells was similar in both groups. The highest number of positive cells was 852 in the control at 14 days, while that in the FGF-2 group was 1667 at 7 days. Therefore, the cell proliferation on the root surface was accelerated by FGF-2, leading to promotion of the connective tissue formation.

### Quantitation of newly formed blood vessels

**Fig 6:**
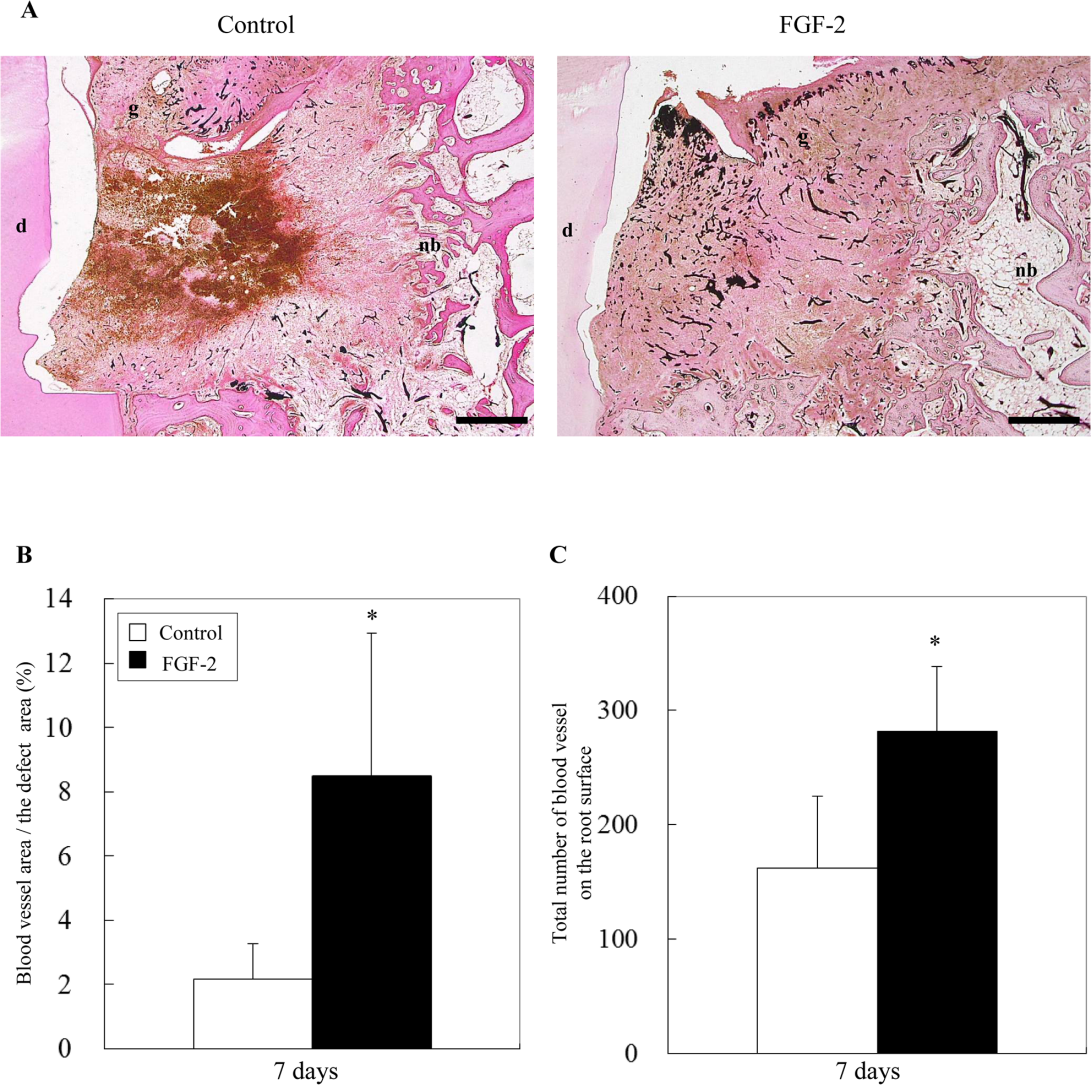
Histomorphometry of angiogenesis at 7 days. (A): Blood vessels were observed by staining the defect with black ink. d, dentin; g, gingival connective tissue; nb, new bone. Scale bar represents 1 mm. (B): The percentage of the blood vessel area in the defect was calculated as the area of black ink / the defect area × 100. (C): The number of blood vessels was counted in the mesio-distal at a 1 mm width of the connective tissue on the root surface. All results are presented as the mean ± SD (n = 4) (*, p <0.05, compared with the control, t-test).

Angiogenesis is clearly essential for the regeneration of periodontal tissue to supply oxygen and nutrients. To quantitate the angiogenic effect of FGF-2 on the periodontal defect, the blood vessel area stained by black ink was measured at 7 days when angiogenesis was most active (Fig 6). Furthermore, we counted the number of blood vessels in the connective tissue on the root surface.

Newly formed blood vessels were observed locally in the control group but entirely in the FGF-2 group (Fig 6A). In the FGF-2 group, the percentage of the blood vessel area in the defect was significantly increased by 4-fold compared with that in the control (Fig 6B). The number of blood vessels in connective tissue on the root surface in the FGF-2 group was twice that in the control (Fig 6C). Therefore, FGF-2 promoted angiogenesis in the periodontal defect.

### Expression of osteogenic genes in the regenerated tissue

**Fig 7:**
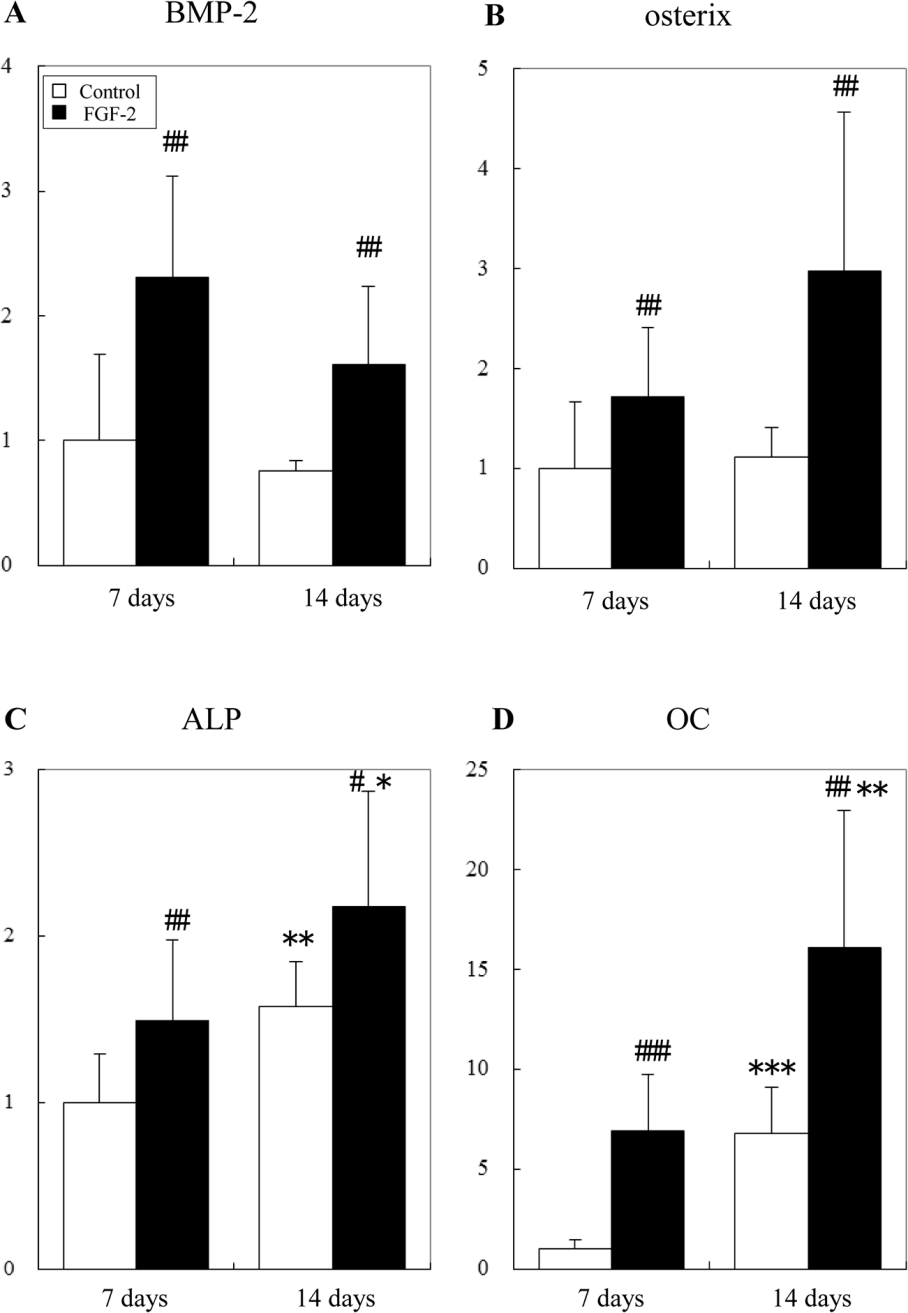
Effect of FGF-2 on the expression of osteogenic genes in the defect. Expression of BMP-2 (A), osterix (B), ALP (C), and OC (D) was examined by quantitative real-time PCR at 7 and 14 days. 18S rRNA was used as an internal control. Y-axis values represent the relative ratio to the mean of the control group at 7 days. All results are presented as the mean ± SD (n = 6) (*, p <0.05; **, p <0.01, compared between 7 days and 14 days; #, p <0.05; ##, p <0.01,compared between the control and FGF-2 group, linear mixed model).

Using quantitative real-time PCR, we examined the effect of FGF-2 on the expression of osteogenic genes in the regenerated tissue of the defect at 7 and 14 days (Fig 7).

BMP-2 and osterix expression was increased significantly in the FGF-2 group compared with that in the control group at 7 and 14 days. Expression of ALP and OC increased with time. This indicates the osteoblastic differentiation was advanced over time. Moreover, the expression of ALP and OC was also increased significantly in the FGF-2 group compared with that in the control group at 7 and 14 days. These results indicate that osteoblastic differentiation in the FGF-2 group preceded more efficiently than that in the control group.

Therefore, FGF-2 may induce the promotion of osteoblastic differentiation via the acceleration of BMP-2 production, resulting in the promotion of bone and cementum formation.

## Discussion

This study presents a detailed qualitative analysis of periodontal regeneration induced by FGF-2 in the early regeneration phase. We examined the action mechanism of FGF-2 in the early formation of regenerated tissues, mainly based on the promotion of cell proliferation that eventually enhances the amount of regenerated periodontal tissues.

Our quantitative analysis indicated that granulation tissue with neovessels formed adjacent to the existing tissue, gradually extended across the bone defect, and was rapidly replaced with new bone (Fig 1). On the root surface, the vascularized connective tissue extended coronally from the existing PDL and formed a new cementum and PDL (Fig 2). This regenerative process was in accordance with our previous findings [11] and included three overlapping stages: the granulation tissue stage, fibrous connective tissue formation stage, and bone formation stage [19]. The regeneration process was similar in control and FGF-2 groups. However, compared with the control, disappearance of the clot, connective tissue formation on the root surface, and new bone formation were significantly promoted by FGF-2 at 7 days. This early formation of regenerated tissues by FGF-2 is critical to maintain the height of gingival tissue at a high level, create a regenerative space, and eventually promote the height of the new bone at 14 days as well as the cementum and PDL at 28 days. In particular, on the root surface, the early formation of connective tissue extending from the existing PDL seems to contribute to inhibiting down-growth of gingival epithelial tissue [4].

These results revealed that the promotion of normal tissue formation induced by FGF-2 at only 7 days leads to enhanced new alveolar bone, cementum, and PDL.

We showed that PCNA-positive cells emerged around the existing bone and PDL, subsequently expanded to the margin, center, and upper zones of the defect, and eventually filled the entire defect (Figs 3 and 5B’–5G’), and that the PCNA-positive cells expressed vimentin (S1 and S2 Figs). These results suggest that the PCNA-positive cells are fibroblastic cells originating from the existing bone marrow and PDL. These fibroblastic cells are thought to contain mesenchymal stem cells from the existing bone marrow and PDL, which can differentiate into osteoblasts and cementoblasts [20–23]. After expansion throughout the entire defect, these fibroblastic cells play an important role in the regeneration of bone, PDL, and cementum [19]. The proliferating cells observed in the existing bone and PDL were similar to those in previous observations [19, 24].

We demonstrated that the number of PCNA-positive cells, fibroblastic cells derived from bone marrow and PDL, was dramatically increased by FGF-2 (Figs 4 and 5H). Various *in vitro* experiments have reported that FGF-2 promotes the migration [25, 26] and proliferation [27] of PDL cells and enhances the proliferation of osteogenic progenitor cells from bone marrow [28–30]. Additionally, the proliferative cells in the FGF-2 group emerged broadly compared with those in the control at 3 days. The peak of proliferative activity in the control group was 14 days, whereas the peak proliferative period in the FGF-2 group was 7 days. Therefore, FGF-2 not only enhanced but also accelerated the proliferative activity of the fibroblastic cells derived from the bone marrow and PDL.

Furthermore, the administrated FGF-2 was distributed in the surface and periphery of the existing bone and PDL, and on the root surface within 3 days (T. Nagayasu-Tanaka et. al., unpublished data). The mRNA expression of endogenous FGF-2 is first detected in immature granulation tissue at 3 days after the periodontal surgery and increases for up to 7 days [11]. FGF-2 up-regulates BMP-2, transforming growth factor-β1, and vascular endothelial growth factor (VEGF) expression in human bone marrow stromal cells [31, 32], and VEGF expression in human PDL stem/progenitor cell lines [33]. Thus, before the increase of endogenous FGF-2 expression, administrated FGF-2 was considered to trigger the regenerative reaction including the cell proliferation and production of other growth factors.

The PCNA-positive cells expanded by FGF-2 were considered to include endothelial cells because FGF-2 is known to facilitate the proliferation of endothelial cells [34]. Additionally, experiments using *in vitro* maintained PDL cells and endothelial cells revealed that FGF-2 stimulated production of VEGF in PDL cells and that FGF-2 and FGF-2-induced VEGF synergistically stimulated angiogenesis [35]. Thus, the area and number of blood vessels were significantly increased by FGF-2 compared with those in the control (Fig 6). In this study, we demonstrated that *in vivo* angiogenesis was promoted by FGF-2 during periodontal healing. Angiogenesis is essential to regenerate tissues by supplying oxygen and nutrients, and is required for successful bone induction during osteogenesis [36].

Thus, these findings revealed that FGF-2 not only promoted the proliferation of fibroblastic cells but also angiogenesis, resulting in the enhancement and acceleration of the new tissue formation at the early regenerative phase.

BMP-2 is an osteogenic cytokine that promotes differentiation of mesenchymal cells into osteoblasts *in vitro* and induces bone formation *in vivo* [37]. Furthermore, BMP-2 controls the expression and functions of osterix [38, 39] and regulates transcription of osteogenic genes such as ALP, type I collagen, and OC [40].

We found that FGF-2 increased the expression of osteoblastic differentiation markers, osterix, ALP, and OC, in the regenerated tissue (Fig 7). Interestingly, the expression of BMP-2 was enhanced by FGF-2 at 7 days when FGF-2 promoted cell proliferation. *In vitro* studies, FGF-2 increased ALP activity, OC production, and bone nodule formation following the enhancements of the proliferation of osteogenic progenitor cells from bone marrow [28–30]. FGF-2 also up-regulated the BMP-2 expression in human bone marrow stromal cells [31]. These results suggest that FGF-2 may increase a specific cell population with high expression of BMP-2, such as BMP-2-producing cells or osteogenic progenitors in the regenerated tissue of the defect. However, further studies are needed to identify the cell population stimulated by FGF-2.

These results suggest that FGF-2 may rapidly induce osteoblastic differentiation at least in part because of BMP-2 production following cell proliferation. This BMP-2 production accelerates the replacement of granulation tissue with new bone, resulting in the promotion of bone formation. Additionally, the increase in BMP-2 concentration as a result of the enhancement of cell proliferation may induce normal regeneration without ankylosis, unlike direct administration of BMP-2, which induces ankylosis [41–43].

The present study revealed facilitatory mechanisms of FGF-2 in periodontal regeneration *in vivo*. First, FGF-2 accelerated and enhanced the proliferation of fibroblastic cells derived from the bone marrow and PDL to create a cell population for new tissue formation. Second, FGF-2 enhanced angiogenesis. Finally, FGF-2 promoted the expression of BMP-2 to facilitate osteoblastic differentiation and bone formation. These findings suggest that, during the initial stages of periodontal tissue regeneration, FGF-2 increases the number of fibroblastic cells including mesenchymal stem cells and promotes angiogenesis. During subsequent healing processes, probably at 3–7 days after application, when FGF-2 activity disappears gradually at the administration site, the fibroblastic cells begin to differentiate into osteoblasts, cementoblasts, and PDL cells, inducing marked periodontal tissue regeneration. We also demonstrated that FGF-2 showed significant superiority over the vehicle alone in terms of the percentage of bone fill in clinical trials [15]. Therefore, based on the multifaceted effects of FGF-2 demonstrated in this study, topical application of FGF-2 to periodontal defects during periodontal surgery would enhance and accelerate the periodontal regeneration.

However, these results in a dog model cannot simply translate to humans because periodontal regeneration in humans is reported to be slower and to a lesser degree compared with beagle dogs. The differences in temporal changes and the extent of the periodontal regeneration are considered to arise because of not only the species difference but also other factors such as smoking habits, coexisting disease including obesity and diabetes, and medical history.

## Supporting Information

S1 Fig Double staining of PCNA and vimentin in the bone defect at 7 days. Representative histological overviews from control (A) and FGF-2 (B) groups are shown in the outside and marginal zones of the defect [Fig 3A (a)]. Double staining of PCNA (blue) and vimentin (brown) in the rectangular areas in A and B are shown in A’ and B’. Photomicrographs at 3, 14, and 28 days are not shown. b, existing bone. Scale bar represents 100 μm.
(TIF)

S2 Fig Double staining of PCNA and vimentin on the root surface at 7 days. Representative histological overviews from control (A) and FGF-2 (B) groups are shown in the defect bottom around the existing PDL [Fig 5A (a)]. Double staining of PCNA (blue) and vimentin (brown) in the rectangular areas in A and B are shown in A’ and B’. Photomicrographs at 3, 14, and 28 days are not shown. b, existing bone; d, dentin; p, existing PDL. Scale bar represents 100 μm.
(TIF)
