# Supplementary figures and images for: Action Mechanism of Fibroblast Growth Factor-2 (FGF-2) in the Promotion of Periodontal Regeneration in Beagle Dogs
Source: PLoS One. 2015 Jun 29;10(6):e0131870. doi: 10.1371/journal.pone.0131870 (PMC4488280; doi:10.1371/journal.pone.0131870)

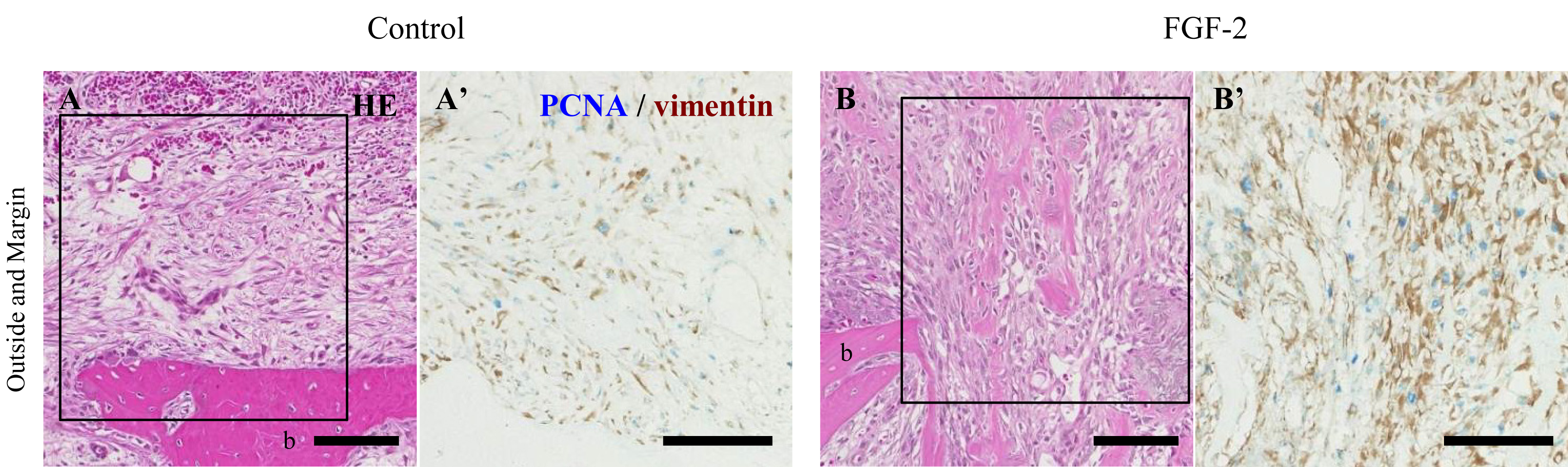

Supplement: S1 Fig — Representative histological overviews from control (A) and FGF-2 (B) groups are shown in the outside and marginal zones of the defect [Fig 3A (a)]. Double staining of PCNA (blue) and vimentin (brown) in the rectangular areas in A and B are shown in A’ and B’. Photomicrographs at 3, 14, and 28 days are not shown. b, existing bone. Scale bar represents 100 μm. (TIF) [file pone.0131870.s001.tif]

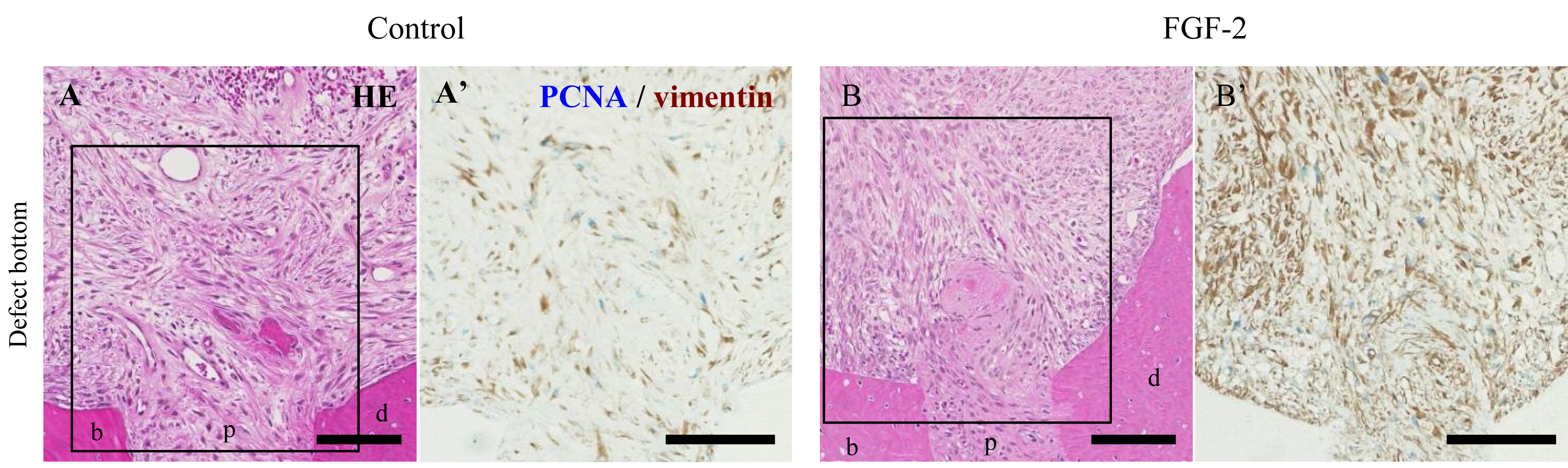

Supplement: S2 Fig — Representative histological overviews from control (A) and FGF-2 (B) groups are shown in the defect bottom around the existing PDL [Fig 5A (a)]. Double staining of PCNA (blue) and vimentin (brown) in the rectangular areas in A and B are shown in A’ and B’. Photomicrographs at 3, 14, and 28 days are not shown. b, existing bone; d, dentin; p, existing PDL. Scale bar represents 100 μm. (TIF) [file pone.0131870.s002.tif]
